# The clot thickens: Autologous and allogeneic fibrin sealants are mechanically equivalent in an *ex vivo* model of cartilage repair

**DOI:** 10.1371/journal.pone.0224756

**Published:** 2019-11-08

**Authors:** Rebecca M. Irwin, Lawrence J. Bonassar, Itai Cohen, Andrea M. Matuska, Jacqueline Commins, Brian Cole, Lisa A. Fortier

**Affiliations:** 1 Nancy E. and Peter C. Meinig School of Biomedical Engineering, Cornell University, Ithaca, New York, United States of America; 2 Sibley School of Mechanical and Aerospace Engineering, Cornell University, Ithaca, New York, United States of America; 3 Department of Physics, Cornell University, Ithaca, New York, United States of America; 4 Research and Development, Arthrex Inc., Naples, Florida, United States of America; 5 Midwest Orthopedics at Rush, Rush University Medical Center, Chicago, Illinois, United States of America; 6 College of Veterinary Medicine, Cornell University, Ithaca, New York, United States of America; University of Connecticut Health Center, UNITED STATES

## Abstract

Fibrin sealants are commonly used in cartilage repair surgeries to adhere cells or grafts into a cartilage defect. Both autologous and commercial allogeneic fibrin sealants are used in cartilage repair surgeries, yet there are no studies characterizing and comparing the mechanical properties of fibrin sealants from all-autologous sources. The objectives of this study were to investigate (i) the effect of fibrinogen and thrombin sources on failure mechanics of sealants, and (ii) how sealants affect the adhesion of particulated cartilage graft material (BioCartilage) to surrounding cartilage under physiological loading. Allogeneic thrombin and fibrinogen were purchased (Tisseel), and autologous sources were prepared from platelet-rich plasma (PRP) and platelet-poor plasma (PPP) generated from human blood. To compare failure characteristics, sealants were sandwiched between cartilage explants and pulled to failure. The effect of sealant on the adhesion of BioCartilage graft to cartilage was determined by quantifying microscale strains at the graft-cartilage interface using an *in vitro* cartilage defect model subjected to shear loading at physiological strains well below failure thresholds. Fibrinogen sources were not equivalent; PRP fibrinogen created sealants that were more brittle, failed at lower strains, and resulted in sustained higher strains through the graft-cartilage interface depth compared to PPP and allogeneic sources. PPP clotted slower compared to PRP, suggesting PPP may percolate deeper into the repair to provide more stability through the tissue depth. There was no difference in bulk failure properties or microscale strains at the graft-cartilage interface between the purely autologous sealant (autologous thrombin + PPP fibrinogen) and the commercial allogeneic sealant. Clinical Significance: All-autologous fibrin sealants fabricated with PPP have comparable adhesion strength as commercial allogeneic sealants *in vitro*, whereas PRP creates an inferior all-autologous sealant that sustains higher strains through the graft-cartilage interface depth.

## Introduction

Fibrin sealants are created by combining fibrinogen and thrombin, and are used in biological repair of nerves, liver, skin, and orthopaedic soft tissues such as articular cartilage[1–5]. Cartilage repair procedures are increasingly performed[6], and commonly utilize allogeneic fibrin sealants to seal chondrocytes[7], live particulated juvenile cartilage[8], dehydrated particulated cartilage[9], or osteochondral allografts/autografts[10] to surrounding native tissue.

Allogeneic sealants such as Tisseel (Baxter Healthcare Corporation, Deerfield, IL, USA) and Evicel (Ethicon, Inc., Somerville, NJ, USA), are pooled from more than one individual. These products have fibrinogen concentrations that are an order of magnitude greater than physiological levels[11,12], and may not be ideal for cartilage repair because they create dense fibrin networks which limits cell migration and matrix deposition[13,14]. Alternatively, all-autologous fibrin sealants can be generated patient-side from blood, or blood that has been centrifuged to generate platelet-rich plasma (PRP) and the waste by-product platelet-poor plasma (PPP).[15,16]. A potential benefit of using an all-autologous compared to an allogeneic fibrin sealant is the presence of growth factors that have the potential to increase matrix synthesis of chondrocytes and improve the quality of repair tissue in vivo[17–20]. However, the mechanical properties of an all-autologous fibrin sealant are unknown.

In contrast to all-autologous fibrin sealants, the mechanical properties of allogeneic sealants have been extensively characterized to have sufficient mechanical strength for adhesion of many non-cartilaginous tissues including the pancreas, liver, and skin[21–25], and their strength is dependent on fibrinogen, but not thrombin concentration[21,26]. While there is an order of magnitude difference in fibrinogen concentration between allogeneic and autologous fibrinogen[11,12], it is unclear what degree of an effect this has on sealant adhesive strength. A few studies have partially investigated an autologous source by combining autologous fibrinogen with human allogeneic thrombin to create autologous/allogeneic hybrid fibrin sealants, and found no difference between the hybrid and allogeneic sealants in tensile strength or stiffness in nerve repair[27], or in compressive behavior[28]. Therefore autologous fibrinogen may be sufficient enough to achieve robust tensile strength, but all-autologous fibrin sealant mechanics have not been investigated.

Even less is known about the adhesive behavior of fibrin sealants in cartilage repair. Pre-clinical studies have examined repair-host tissue failure after a cartilage repair procedure, but adhesive strength was attributed to new tissue and did not consider the fibrin sealant[19,29–34]. Additionally, several of these *in vitro* studies used xenogeneic fibrinogen and thrombin sources. As such, these studies do not provide an assessment for the ability of fibrin sealants to adhere to native cartilage tissue from allogeneic or autologous sources at the time of surgery.

To address these knowledge gaps, this study compared various fibrinogen:thrombin formulations to determine (i) failure mechanics of fibrin sealants as a function of fibrinogen concentration and thrombin source, and (ii) the effect of different sealant sources on adhesion of BioCartilage (Arthrex, Inc., Naples, FL, USA), a hypothermically dehydrated, particulated human articular cartilage graft material, to cartilage. The results of this study were expected to provide clinically important information that could be immediately translated to the clinic regarding the choice of fibrin sealant in cartilage repair procedures by comparing the mechanical property and adhesion characteristics of all-autologous to allogeneic fibrin sealants.

## Materials and methods

### Ethics statement

An IRB or ethics committee approval was not required as the human blood was purchased from a third party company (HemaCare Bioresearch Products, Van Nuys, California, United States of America). Additionally, an IACUC or ethical board approval was not required as animal tissue was purchased from a third party (Gold Medal Packing, Rome, New York, United States of America).

### Fibrin sealant production

Six sealants were evaluated for mechanical properties (Table 1). Two thrombin sources (1. allogeneic, 2. autologous from PPP) and three fibrinogen sources (1. allogeneic, 2. autologous/PRP, and 3. autologous/PPP) were used. All sealants were formed using a dual-ejection syringe where thrombin and fibrinogen were mixed at a 1:1 ratio. Allogeneic source: Allogeneic thrombin and fibrinogen were purchased (Tisseel), Autologous source: Autologous thrombin and two autologous fibrinogen sources (PRP and PPP) were generated from blood with 10% ACD-A purchased from HemaCare Bioresearch Products (Van Nuys, CA). Blood (n = 13) was used within 24 hours of venipuncture, and was separated into PRP and PPP using the Angel System (Arthrex, Inc). Autologous thrombin was created using PPP in the Thrombinator^TM^ System (Arthrex, Inc.) per manufacturer’s instructions. Briefly, 4 mL of PPP was re-calcified with 10% CaCl_2_ in the device and allowed to clot. At the time of use, PPP and CaCl_2_ were added again, and the activated plasma was withdrawn through an 18 μm filter.

**Table 1:** Fibrin sealants evaluated for failure properties.

Sealant	1	2	3	4	5	6
**Thrombin**	**Allogeneic**	**Allogeneic**	**Allogeneic**	**Autologous (PPP Source)**	**Autologous** **(PPP Source)**	**Autologous** **(PPP Source)**
**Fibrinogen**	**Allogeneic**	**Autologous (PPP)**	**Autologous (PRP)**	**Allogeneic**	**Autologous (PPP)**	**Autologous (PRP)**

Six sealants composed of allogeneic (black), autologous blood and plasma (blue), or mixtures of the two were tested.

### Fibrinogen quantification

To understand the contribution of fibrinogen concentration to fibrin sealant mechanics, samples were assayed for fibrinogen concentration using commonly used methods in clinical pathology laboratories. Blood, PRP, and PPP from each patient (n = 13) were assayed for fibrinogen concentration using the Clauss method[35] in an automated coagulation instrument (STACompact, Diagnostica Stago, Parsippany, NJ) using a human thrombin reagent (Fibrinogen-5, Diagnostica Stago) and a human fibrinogen standard (Unicalibrator, Diagnostica Stago) according to the manufacturer’s instructions.

### Thromboelastography (TEG) and thrombin clotting time (TCT) assays

Thromboelastography (TEG) and thrombin clotting time (TCT) assays were performed to determine if these measures, which can be performed at the time of surgery, could serve as predictors of fibrin sealant mechanics for cartilage adhesion. TEG provides a measure of clot mechanical properties[36,37] and was performed on PPP and PRP samples (n = 3). TCT[38] was performed for both allogeneic and autologous thrombin sources (n = 5–6, detailed methods can be found in supplemental information file).

### Adhesive strength–pull-apart tests

**Fig 1:**
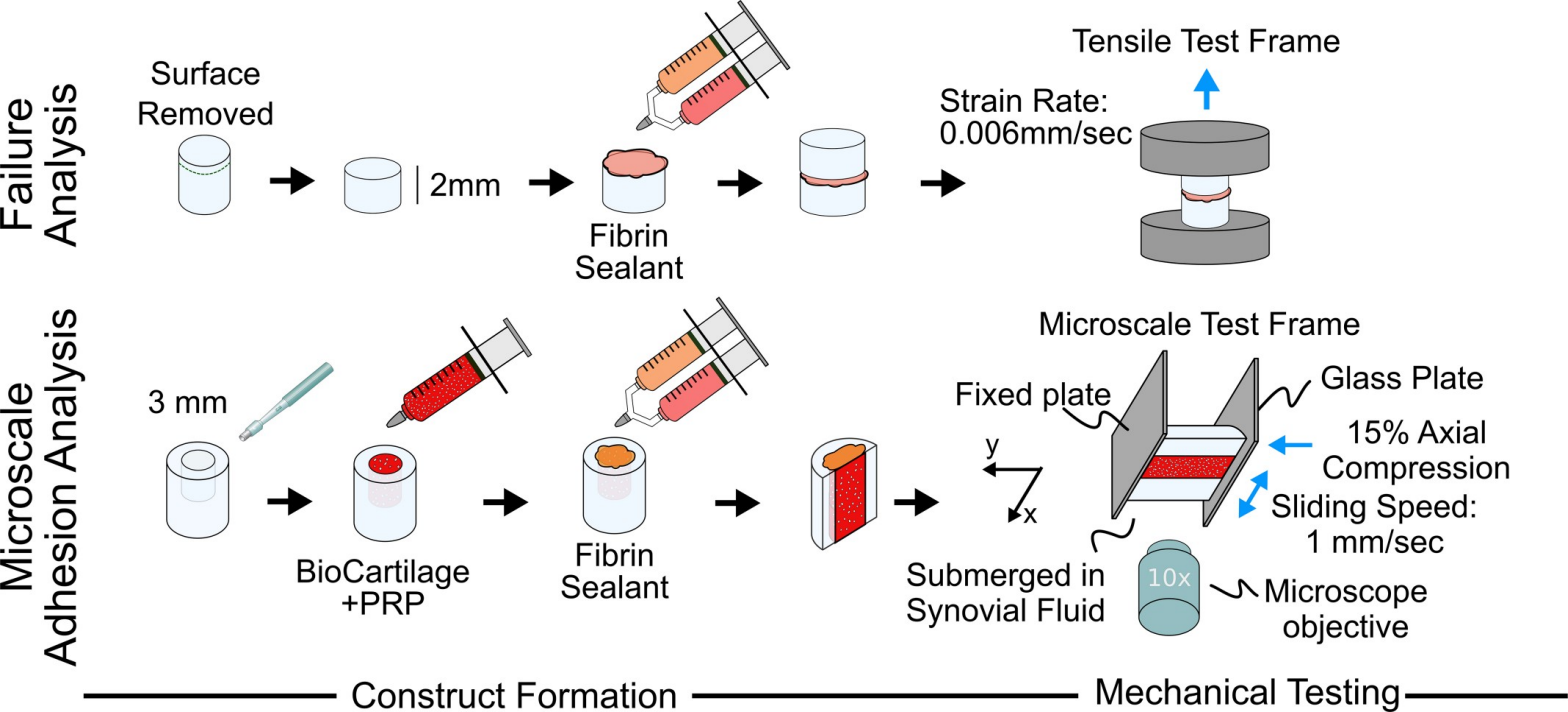
Mechanical analyses flow chart. Articular cartilage cylinders were harvested from neonatal bovids. Failure Analysis: 500 μm was removed from the surface and a fibrin sealant was used to adhere two cylinders together. Sandwich constructs were pulled to failure. Microscale Adhesion Analysis: 3mm core was removed, filled with BioCartilage and PRP mixture, and capped with a fibrin sealant. Constructs were bisected, fluorescently stained, and mounted onto a microscale test frame. Images were captured during shear loading and used to calculate microscale strains at repair interface.

A pull-apart test was used to assess the Young’s Moduli and adhesion strength via failure properties of fibrin sealants sandwiched between cartilage explants (Fig 1, top row)[31,32], to determine if the sealants can withstand physiologic stresses and strains experienced during compression and shear loading. Cartilage was harvested from the patella-femoral groove of 1–3 day old bovids (Gold Medal Packing, Rome, NY, USA) and stored at -20°C until testing. Explants 6 mm in diameter were created with a biopsy punch, and the first 0.5 mm of the articular surface was removed to expose the sub-surface tissue, mimicking the debrided region where in surgery, the fibrin sealants would be used to adhere a graft material to the edges of native surrounding cartilage. Explants were sectioned to 2 mm thick, and sandwich constructs were formed by adhering two explants together using one of 6 sealants described in Table 1. Sandwich constructs were mounted onto an uniaxial test frame (ElectroForce 3100, TA Instruments, New Castle, DE, USA) and pulled to failure at a strain rate of 0.006 mm/sec with force and displacement data collected in real time (n = 8–11 constructs per sealant).

Sealant thickness was determined optically from images taken before mechanical testing and were used to calculate engineering strain. From the stress vs. strain curves, Young’s Modulus, ultimate tensile stress and strain, and toughness were determined. Young’s Moduli measurements were taken at the first linear region of the curve before significant plastic deformation was observed. Ultimate tensile stress and strain were determined by finding the maximum stress and corresponding strain in the stress vs strain curve. Sealant toughness was calculated as the area under the stress vs strain curve.

To further understand the specific role of fibrinogen in the mechanics of fibrin sealants, PPP and PRP were diluted up to 10-fold with PBS to produce a range of concentrations from 7.5 mg/dL to 8700 mg/dL used in pull apart experiments. Fibrinogen values for diluted samples that had concentrations between 0–15 mg/dL, were analyzed to have a concentration of 7.5 mg/dL. Data for allogeneic fibrinogen sealants were analyzed at the median of the reported concentration (8700 mg/dL)[11].

### Ex vivo cartilage repair model

A cartilage repair model was used to examine the effect of fibrin sealant on adhesion of BioCartilage to surrounding, native cartilage under shear loading (Fig 1: bottom row). Cartilage was harvested from the patella-femoral groove of 1–3 day old bovids and sectioned into 6 mm diameter by 2 mm thick cylinders. A 3 mm core was removed from the center of each cylinder and packed with a BioCartilage:PRP mixture to fill 90% of the 3 mm core, leaving ~0.2 mm above the packed repair for application of a fibrin sealant as is performed clinically[9,39,40]. One of the 5 fibrinogen:thrombin sealant combinations excluding sealant #4 in Table 1 were used to complete the constructs. Sealant #4 was not used in microscale analyses because it had equivalent results to both the purely allogeneic sealant and the purely autologous sealant with PPP fibrinogen. Additionally, it is unlikely that autologous thrombin would be combined with an allogeneic fibrinogen source in a surgical application because it would incorporate the expenses of both autologous and allogeneic products.

### Strain quantification at repair interface under dynamic loading

The microscale strains of constructs were obtained using a modified version of previously established protocols[41–43]. Briefly, constructs were bisected into hemi-cylinders and fluorescently stained with 5-dichlorotrianzinyl-aminofluorescein (Molecular Probes, Grand Island, NY, USA) for 1 hour followed by a 20 minute rinse in PBS. Constructs were mounted onto a tissue deformation imaging stage (TDIS) where the deep zone was adhered with cyanoacrylate to a fixed back plate and submerged in equine synovial fluid. The TDIS was mounted on an inverted Zeiss LSM 510 5 live confocal microscope and imaged using a 488nm laser. Constructs were axially compressed at a physiologic level by 15% via a glass plate and allowed 30 minutes to stress relax[44]. After stabilization, the glass plate was slid parallel to the abutting construct surface at an oscillating rate of 1mm/sec while images were captured.

**Fig 2:**
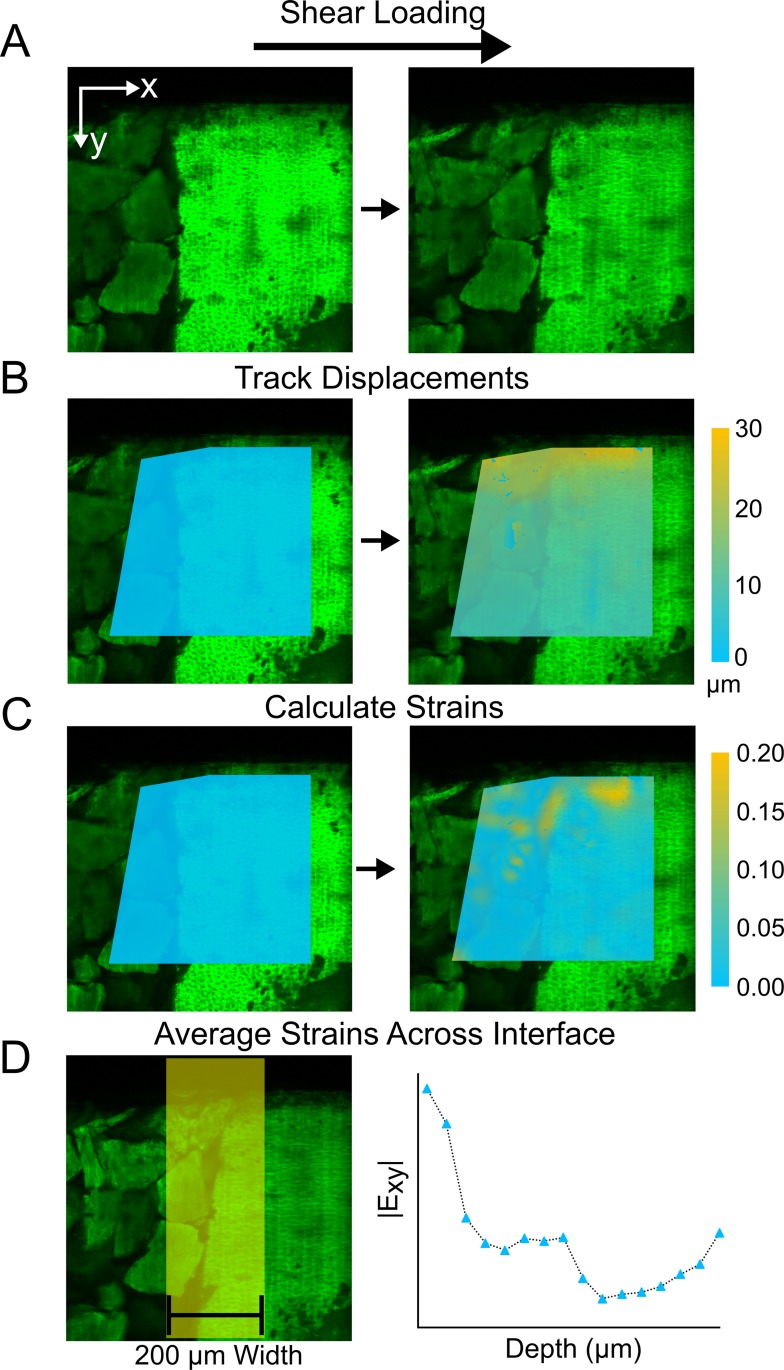
Repair interface strain analysis. Fluorescent confocal images with sealant and BioCartilage repair on the left of the interface and healthy tissue on the right. Under shear loading, the repair and healthy tissue are displaced but remain intact (A). Displacements were tracked using Ncorr in Matlab in drawn region of interest (B). Heat maps of displacements are plotted on the fluorescent images showing highest displacements in the x direction at the surface of the construct for both the repair and healthy tissue. Strains were calculated from displacements (C). Strain magnitudes were averaged across a 200 μm width interface region in the x direction and plotted as a function of depth (D).

Confocal videos were analyzed to extract local deformations and strains. Videos were processed using Ncorr, an open source 2D-digital image correlation (DIC) software implemented in MATLAB (window size 74 μm, grid spacing 7.4 μm, Fig 2A)[45]. A least squares plane was fit on a subset of displacement data (window size 51.8 μm) and the strains were found as a solution of an over-constrained system of equations (Fig 2B). Depth-dependent strain data were calculated from displacements (Fig 2C) and averaged across a 200 μm wide region at the interface between the BioCartilage scaffold repair and healthy tissue (Fig 2D). In a continuous material, tissue axial (E_xx_) strains would be zero under shear loading in the E_yx_ direction. Additionally, orthogonal shear (E_xy_) strains would vary with depth for articular cartilage due to natural variations in composition and material properties[41]. Therefore any non-zero axial (E_xx_) would describe the repair peeling apart from the adjacent native tissue, and divergent shear (E_xy_) strains from native tissue would describe sliding motion at the interface of repair and native tissue. These axial and shear strain magnitudes describe motion of the repair at the interface, indicating the degree of attachment to surrounding native cartilage and predicting mechanisms for repair displacement or failure.

To better understand the spatial variation in local strains through the tissue depth, each sample was fit to an exponential decay equation of the formula: *y* = (*y*_0_−*plateau*)*e^−kx^*+*plateau* (y_0_: strain value at surface where depth is 0 μm; plateau: strain value at infinite depths; K: rate constant, S1 Fig). One sample did not converge to an exponential fit as strains were consistent and below 0.03 for all depths and was excluded from fit parameter comparisons. Our analysis used these strain measurements to assess differences in movement at the interface between sealants.

### Statistical analyses

Fibrinogen concentrations were compared between blood, PPP, and PRP using a two-way ANOVA with source and patient as fixed factors. TEG parameters were compared between PPP and PRP using a paired t-test. TCT assay results between allogeneic and autologous thrombin sources were compared using a t-test. From pull apart experiments, outcome measures of Young’s Modulus, ultimate tensile stress, ultimate tensile strain, and toughness were compared using a linear mixed-effects model and a Tukey multiple comparison with sealant as a fixed factor and a random effect of day the sealants were tested since each patient sample was tested on a different day. Axial and shear strains at the interface of the in vitro cartilage repair model were compared by fitting one phase decay functions to each sample. Function parameters were compared using a two-way ANOVA with sealant and patient as fixed factors. The ANOVA was followed by a Tukey multiple comparisons test. For pull apart results and strain exponential decay fit parameters, Box-Cox power transformations were used to appropriately transform data if residuals were not normally distributed (transformations specified in supplemental materials). For all statistical tests, a p-value of less than 0.05 was considered statistically significant.

## Results

### PPP has highest fibrinogen concentration of autologous sources and achieves slower clot in TEG analysis

**Fig 3:**
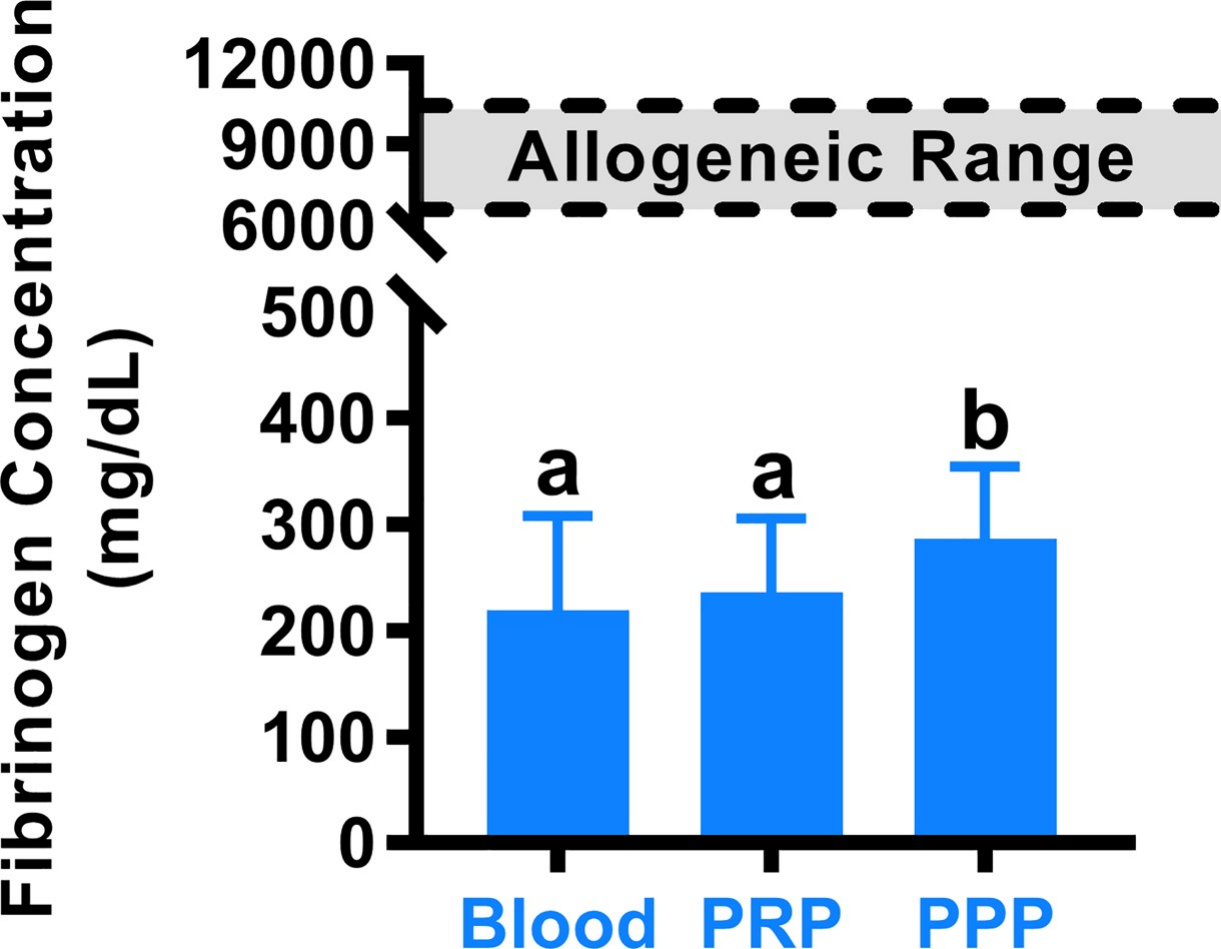
Fibrinogen concentrations for autologous sources. Fibrinogen sources with different letters were statistically different. Reported concentration range for the allogeneic fibrinogen source (Tisseel) was 6,700–10,600 mg/dL and is represented by shaded bar on graph.

PPP had significantly higher fibrinogen concentration compared to PRP (p<0.05) and blood (Fig 3, p<0.01), which were not different from each other (p = 0.33). Allogeneic fibrinogen was too viscous for analysis, so the manufacturer reported fibrinogen concentration of 67–106 mg/mL was used for analyses.

PPP had a longer time to initial fibrin formation (R, p<0.05), longer time to achieve clot strength (K, p<0.05), slower rate of clot formation (angle, p<0.05), lower maximal clot strength (MA, p<0.05), and lower tensile properties over time (G, (p<0.05) compared to PRP (S1 Table, paired t-tests). There were no differences in lysis times (LY30, LY60, and CLT, p>0.24).

### Allogeneic thrombin activity is two orders of magnitude greater than autologous source

Allogeneic thrombin induced clotting faster than autologous source thrombin (p<0.001, t-test) with an estimated allogeneic thrombin activity of 770±84 IU/mL (mean ± SD). This value is similar to the manufacturer’s reported value of 400–625 IU/mL. In contrast, the thrombin activity of autologous thrombin was two orders of magnitude lower at 3.8±2.7 IU/mL.

### All-allogeneic and all-autologous sealants have similar failure properties

**Fig 4:**
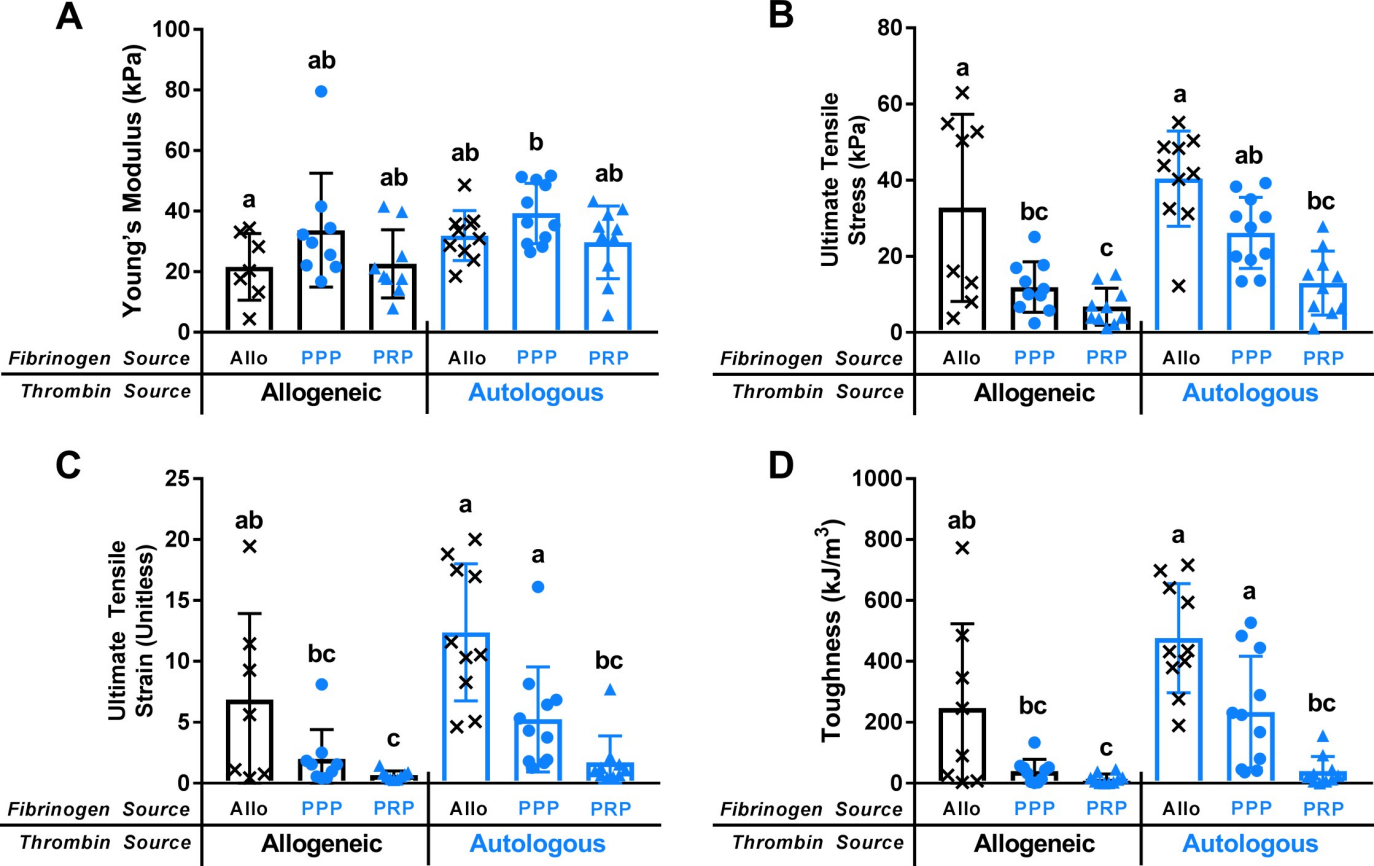
Young’s Modulus and failure properties of fibrin sealants. Young’s Modulus (A) and failure outcomes of ultimate tensile stress (B), ultimate tensile strain (unitless) (C), and toughness (D) for fibrin sealants. Thrombin source denoted by bar outline color (allogeneic: black; autologous: blue) and fibrinogen source by data point color and shape (x: allogeneic; circle: PPP; triangle: PRP). Fibrinogen sources on x-axis. Sealants with different letters were statistically significant.

Differences in Young’s Moduli were only observed between the all-allogeneic sealant and autologous PPP + thrombin fibrin sealant (Fig 4A, p<0.05). The failure properties for the all-allogeneic and the all-autologous sealant with PPP fibrinogen were not different (Fig 4B, 4C and 4D).

### Fibrinogen and thrombin sources affect sealant toughness and failure strain

The all-autologous sealant fabricated from PPP fibrinogen had superior failure properties to those fabricated from PRP fibrinogen regardless of thrombin source. PPP fibrinogen with autologous thrombin withstood strains 3–9 times higher before failing compared to PRP fibrinogen sealants (Fig 4C). Most notably, allogeneic fibrinogen and PPP fibrinogen with autologous thrombin sealants had toughness values an order of magnitude greater than the sealants created with PRP fibrinogen, indicating that PRP fibrinogen sealants were significantly more brittle (Fig 4D).

Despite a thrombin activity two orders of magnitude below allogeneic thrombin, autologous thrombin created superior sealants that failed at higher strains (Fig 4C, p<0.05) and had higher toughness (more ductile, Fig 4D, p<0.01) when combined with PPP fibrinogen.

### Fibrinogen concentration improves fibrin sealant failure properties

**Fig 5:**
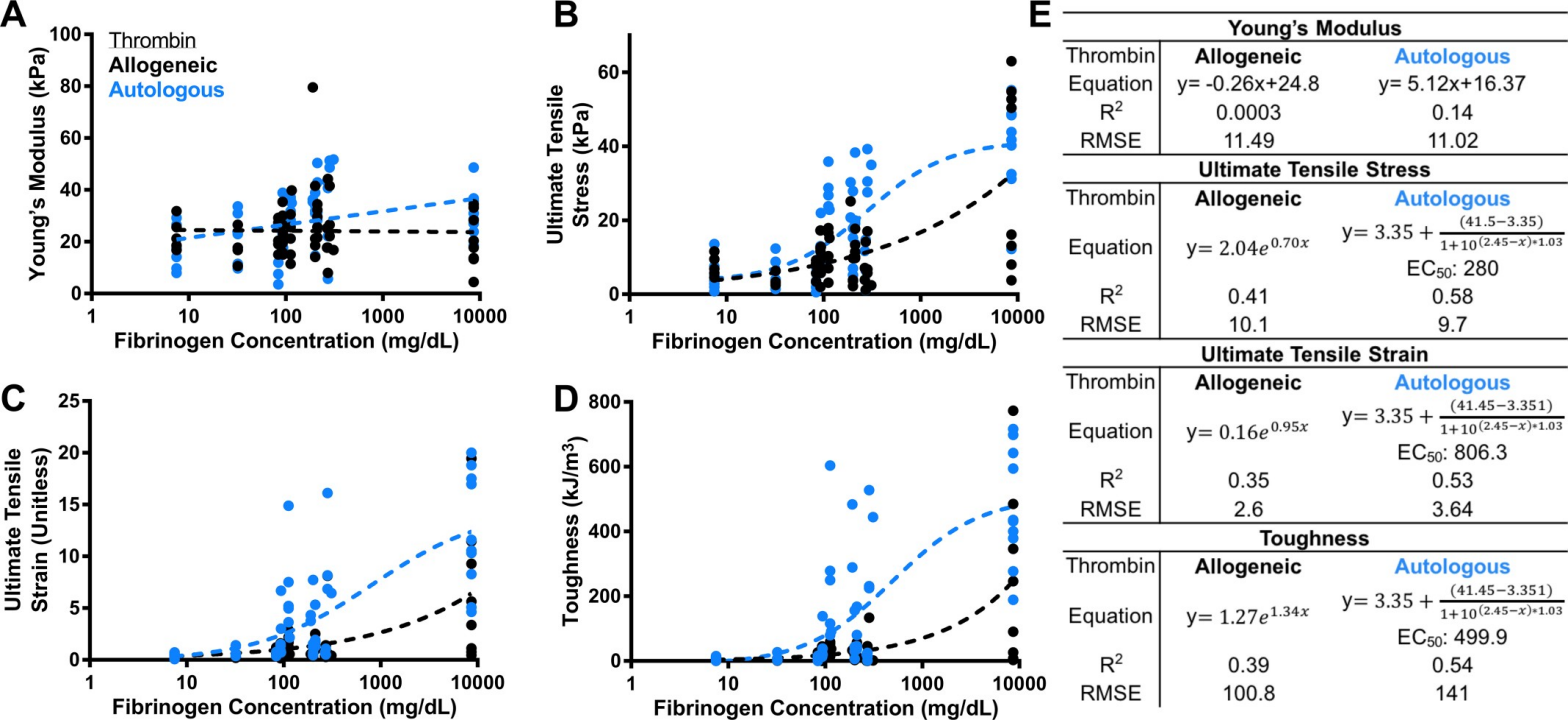
Failure properties improve with increased fibrinogen concentration. Young’s Modulus (A) and failure properties (B-D) plotted as functions of fibrinogen concentration. Strains are unitless. Fibrin sealants fabricated from allogeneic thrombin shown in black, and those fabricated from autologous thrombin shown in blue. Each individual sample represented by a data point. Fit parameters and goodness of fit measurements are summarized in panel (E).

Young’s Modulus was mostly insensitive to fibrinogen concentration for all sources (Fig 5A). Increased fibrinogen concentrations improved fibrin sealant failure metrics regardless of source (Fig 5B, 5C and 5D). Thrombin sources exhibited different behaviors within fibrinogen concentrations tested where autologous thrombin followed a dose-response fit (R^2^ = 0.53–0.58), and allogeneic thrombin had improved failure properties following an exponential growth fit (R^2^ = 0.35–41). Even at diluted values down to 7.5 mg/dL of fibrinogen, fibrin clots formed that were able to adhere cartilage explants together. EC_50_ values for the dose-response curve fits indicate the fibrinogen concentration at which half the maximum achievable failure property value is reached. All EC_50_ values were on the order of magnitude of undiluted autologous sources (Fig 5E).

### Interface strains are highest at surface region of construct

**Fig 6:**
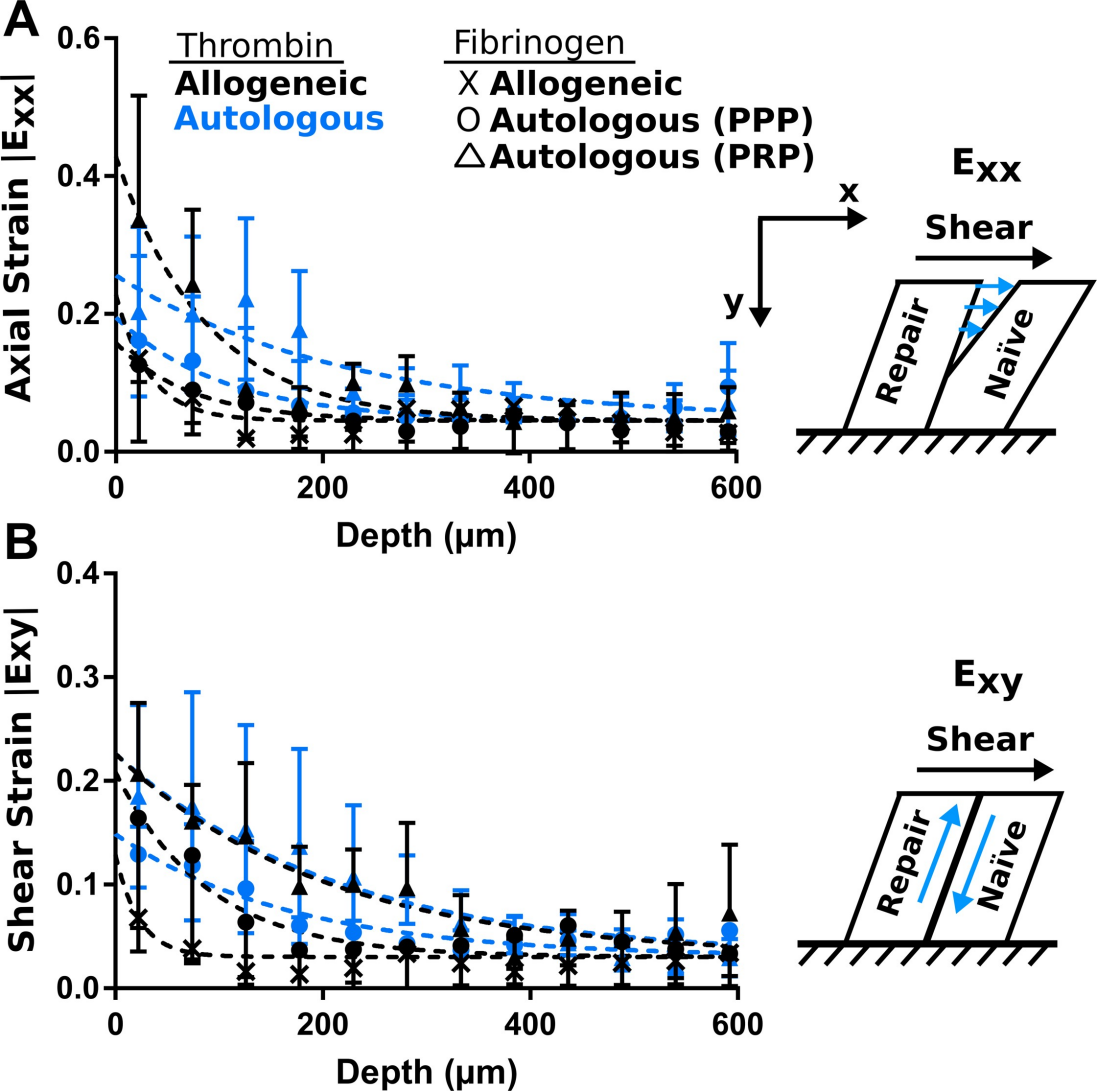
Strains are highest at construct surface and similar between sealants. Axial strains (a) and shear strains (b) are highest at construct surface and decrease with depth. Higher strains indicate pulling apart (axial, E_xx_) or sliding (shear, E_xy_) at the interface. Averages of individual samples plotted ± standard deviations (n = 2–4 per sealant). Exponential fits shown by dashed lines for strain averages. Strains are unitless.

Axial and shear strains followed an exponential decay behavior with construct depth for all sealants (Fig 6A and 6B, n = 2–4). As both axial and shear strains were highest at the construct surface for all sealants, this is the region where failure would most likely occur. Surface axial (E_xx_) strains decreased by a factor of 4 from the surface to plateau values for all sealants. Similarly, shear (E_xy_) strains decreased by a factor of 3–3.5 from the surface plateau values for all sealants. All strains were at least 1.6 times lower than failure thresholds.

### Interface strains dissipate faster in PPP fibrinogen sealants compared to autologous PRP sealant

The rate (K) at which strains decayed from the surface to the plateau values were different between sealants (S2 Fig; p<0.05). Both the all-allogeneic and all-autologous using PPP fibrinogen sealants had higher decay rates for axial strains compared to the all-autologous sealant fabricated with PRP fibrinogen (S2 Fig; p<0.05). Additionally, the allogeneic thrombin and PPP fibrinogen had a higher decay rate compared to the all-autologous sealant with PRP (S2 Fig; p<0.05). A higher decay rate is desirable because it reflects that strain magnitudes decrease at earlier depths instead of being sustained for greater depths in the tissue. These results indicate that the autologous thrombin + PRP fibrinogen sealant is inferior as it sustains higher strains at greater depths as the decay rate is lower for both axial and shear strains. Maximum strains at the construct surface (Y_0_) and minimum strain plateau values were not different between sealants for axial strains (S2 Fig; p>0.68) or shear strains (S2 Fig; p>0.40).

## Discussion

The objectives of this study were to assess the mechanical properties of two clinically available fibrin sealant sources for cartilage repair. Our results indicate that the all-autologous sealant with PPP fibrinogen had superior bulk failure mechanics and lower sustained interfacial graft-cartilage strains compared to the all-autologous sealant with PRP fibrinogen, but was not different from the allogeneic sealant (Tisseel) for either mechanical assessments. The all-autologous sealant with PPP may have achieved better adhesion between the BioCartilage graft and adjacent cartilage compared to PRP as it takes longer to form a clot, allowing the sealant to percolate deeper into the repair and provide more stability to the graft. As such, the all-autologous sealant with PRP resulted in weaker adhesion demonstrated by sustained higher strains through the tissue depth. Such data suggests PPP should be used in autologous fibrin sealant fabrication instead of PRP. Additionally, all-allogeneic and all-autologous sealants provide mechanically similar graft adhesion to articular cartilage, and therefore the biological ramifications could be considered when deciding which source to use. Autologous components contain lower fibrinogen and thrombin concentrations compared to allogeneic sources, and this could lead to improved cell migration and matrix deposition[46–50]. Our results demonstrate that the benefits of autologous source components can be appreciated without sacrificing mechanical performance of the fibrin sealant.

Using an in vitro defect model we assessed strains at the interface of repair describing both the nature of and magnitude of motion at the interface of repair and healthy tissue. Motion at the interface of repair may lead to graft failure[51], and therefore the ability to quantify motion by assessing strains provides an understanding of where failure is more likely to occur. Our results show that there was no difference in peak strains between sealants, but strains in constructs capped with the autologous thrombin + PRP fibrinogen sealant were sustained at greater depths. As such, the cartilage repair is pulled apart from healthy tissue at higher magnitudes for greater spans along the interface. However, for all sealants the strains at the interface were at least 1.6 times lower than the failure strains determined from the pull-apart tests, confirming their capability of adhering BioCartilage to articular cartilage under physiologic loading conditions. The shear strain magnitudes at the interface of repair were supraphysiologic compared to strains in intact healthy tissue[52], but it is unknown how strains are altered at the interface of a defect *in vivo*. While our sample size for these analyses was low (n = 2–4 per sealant) and should be repeated to obtain more confident values of strain magnitude at the repair interface, we were able to assess that all outcomes measures of strain were below failure thresholds. This confirms that under shear loading, all sealants adhered a particulated cartilage repair to surrounding native cartilage tissue.

Our results defining relationships between fibrinogen concentration and mechanical strength of fibrin sealants are consistent with previous studies, as fibrinogen concentration has been correlated with fibrin clot strength[21,26]. PPP fibrinogen when combined with autologous thrombin created a sealant mechanically similar to the allogeneic source sealant (Fig 3). Therefore, PPP contains sufficient fibrinogen to create robust sealants with mechanically similar failure properties as allogeneic fibrinogen and at an order of magnitude lower concentration. High fibrinogen concentrations have been shown to create dense fibrin glues that inhibit cell migration and matrix deposition[14,48,53]. As such, the lower fibrinogen concentrations seen with autologous sources like PPP may promote superior repair compared to allogeneic fibrinogen without compromising mechanical performance.

Thrombin concentration has also been investigated as a predictor of fibrin clot strength, with results showing no correlation[26]. We report corroborating results showing no difference in mechanical outcomes between thrombin sources despite thrombin activities that vary by two orders of magnitude between allogeneic and autologous sources. Furthermore, higher thrombin concentrations could have negative biological consequences affecting cell viability, morphology, and proliferation[49,50]. The relatively low thrombin activity in autologous sources may therefore provide a more suitable environment for cartilage repair.

Interestingly, our TEG data results comparing autologous fibrinogen sources did not mirror our strength analyses for sealant failure properties. PRP had higher clot strength than PPP in TEG measurements, demonstrating the procoagulant properties of platelets in promoting fibrin formation and effects of clot retraction in bonding the TEG pin to the reaction cup. Nevertheless, in the pull-apart tests PRP created sealants more brittle and with lower failure strain compared to PPP when combined with autologous thrombin. Additionally, higher strains at the repair interface were sustained at greater depths for the autologous thrombin with PRP fibrinogen compared to the sealants with PPP and allogeneic fibrinogen sources. These results indicate that TEG measurements provide strength analyses for individual fibrinogen components, but these properties may not reflect sealant properties when fibrinogen components are mixed with thrombin in an in vitro repair experiment model. As such, TEG is not recommended as a clinical measurement to predict individual patient fibrin clot strength at the time of surgery.

The mechanical properties of Tisseel (allogeneic source) have been evaluated in a variety of experimental designs[21,24] with wide ranges in reported values. Tensile strength reports vary from 0.775 Pa in a liver adhesion experiment[23] to 29 kPa in a uniaxial test of the sealant itself[25], while Young’s Modulus reports span an order of magnitude from 15 kPa to 300 kPa[25,31]. Our result for tensile strength (32.7 kPa) and Young’s Modulus (20.7 kPa) fall within the range of values, but high variability in our failure outcome metrics was observed for all fibrin sealants evaluated in the pull apart tests. Due to the small extrusion volume needed for these experiments and the physical challenge of administering the sealant, the variability could be attributed to the application technique. Precise extrusion of small volumes of a thrombin and fibrinogen source through a dual-ejection syringe is challenging and inconsistent, requiring a uniform force to be applied across two syringes. Alterations could be made to the dual-extrusion system design to improve repeatability and ease of use of fibrin sealants.

There are several limitations to consider when interpreting these data. While our analysis included the lower limits of fibrinogen concentration values via dilution, fibrinogen concentrations exceeding that from commercially available sources were not tested. Therefore, the fitted relationships between fibrinogen concentration and failure mechanics for allogeneic thrombin could follow a dose-response fit and plateau at higher fibrinogen concentrations, but this is outside the scope of clinically available fibrinogen sources.

We assessed microscale strains at the interface of repair immediately following construct formation using an in vitro system that does not capture joint composition and mechanical loading patterns. Additionally we performed this study using healthy neonatal bovine cartilage which differs structurally and compositionally from adult human tissue. We chose this source because it is a reproducible model that allows for the identification of differences between fibrin sealants independent of tissue condition. The goal of this study was to examine deformations in cartilage and BioCartilage during sliding, which primarily produces shear strains in these tissues. Previous studies have shown that the shear modulus of adult human and neonatal bovine articular cartilage are similar quantitatively and qualitatively[42,54]. Further, frictional behavior during sliding is also similar between these sources[54,55] and such frictional properties are predictive of clinical efficacy[56]. Our experimental timescale was short enough to ignore any degradation of the fibrin clot from enzymes found in native synovial fluid[57] and the applied axial compressive strains were within physiological ranges[44]. However we do not know the long term decay properties of these sealants or how the sealants would be maintained *in vivo*. Our results still allow for the comparison of strain magnitudes among sealants, and give an understanding of the length scale of which fibrin sealants affect the strains at the interface of repair. In addition, no analysis was performed on mechanical parameters of a BioCartilage-subchondral bone interface which will be the subject of future study.

In this study, allogeneic and autologous sources for fibrin sealants were extensively characterized. Fibrinogen concentration predicted mechanical performance, with autologous PPP providing sufficient fibrinogen for robust adhesion to articular cartilage. Importantly, no difference was seen between all-autologous sealant with PPP and the commercial allogeneic sealant in failure mechanical properties or microscale strain magnitudes when adhering a repair to healthy cartilage. Our results support the clinical use of autologous source sealants to minimize patient exposure to foreign proteins or pathogens without compromising mechanical performance.

## Supporting information

S1 Fig Individual sample exponential fits for strains through construct depth. Column header denotes strain and row header denotes sealant group (thrombin + fibrinogen). Exponential decay fits plotted as lines through data points. Color denotes the patient and date tested. Patient 3 in allogeneic thrombin + PPP fibrinogen sealant group was only sample to not converge to an exponential fit as strains were consistent and below 0.03 for all depths. Average strain across 200 μm width region at repair interface ± standard deviations.
(TIF)

S2 Fig Exponential decay fit parameters for fibrin sealants. Axial (a-c) and shear strain (d-f) exponential fit parameters from individual samples for all sealants (y_0_: strain value where depth is 0 μm, plateau: strain value at infinite depths, K: rate constant). The autologous thrombin + PRP fibrinogen sealant had significantly lower K values (rate constants) for both axial and shear strains. Letters denote statistical significance where sealants with same letters were not statistically different and sealants with different letters were statistically significant.
(TIF)

S1 File Graft-cartilage repair interface under shear loading. Fluorescent graft (left) and articular cartilage (right) subjected to shear loading while bathed in synovial fluid. Graft-cartilage interface in center of video demonstrated peeling (axial strain, E_xx_) and sliding (shear strain, E_xy_).
(AVI)

S1 Supporting Information Thromboelastography, Thrombin Clotting Time Assay, and Box Cox Transformation Methods. Detailed methods for thromboelastography and thrombin clotting time assay measurements conducted in this study. Additional information on how box cox transformations were performed on data for statistical analyses.
(DOCX)

S1 Table Thromboelastography results for autologous fibrinogen sources. The TEG parameters generated by the instrument software include initiation (R) and rate of fibrin formation (K, angle), maximal clot strength (MA), tensile properties over time (G), extent of lysis at 30 minutes and 60 minutes (LY30, LY60) and time to maximal lysis (CLT). Bolded p-values are statistically significant. Means ± standard deviations (n = 3).
(DOCX)
