# Supplementary figures and images for: The clot thickens: Autologous and allogeneic fibrin sealants are mechanically equivalent in an ex vivo model of cartilage repair
Source: PLoS One. 2019 Nov 8;14(11):e0224756. doi: 10.1371/journal.pone.0224756 (PMC6839864; doi:10.1371/journal.pone.0224756)

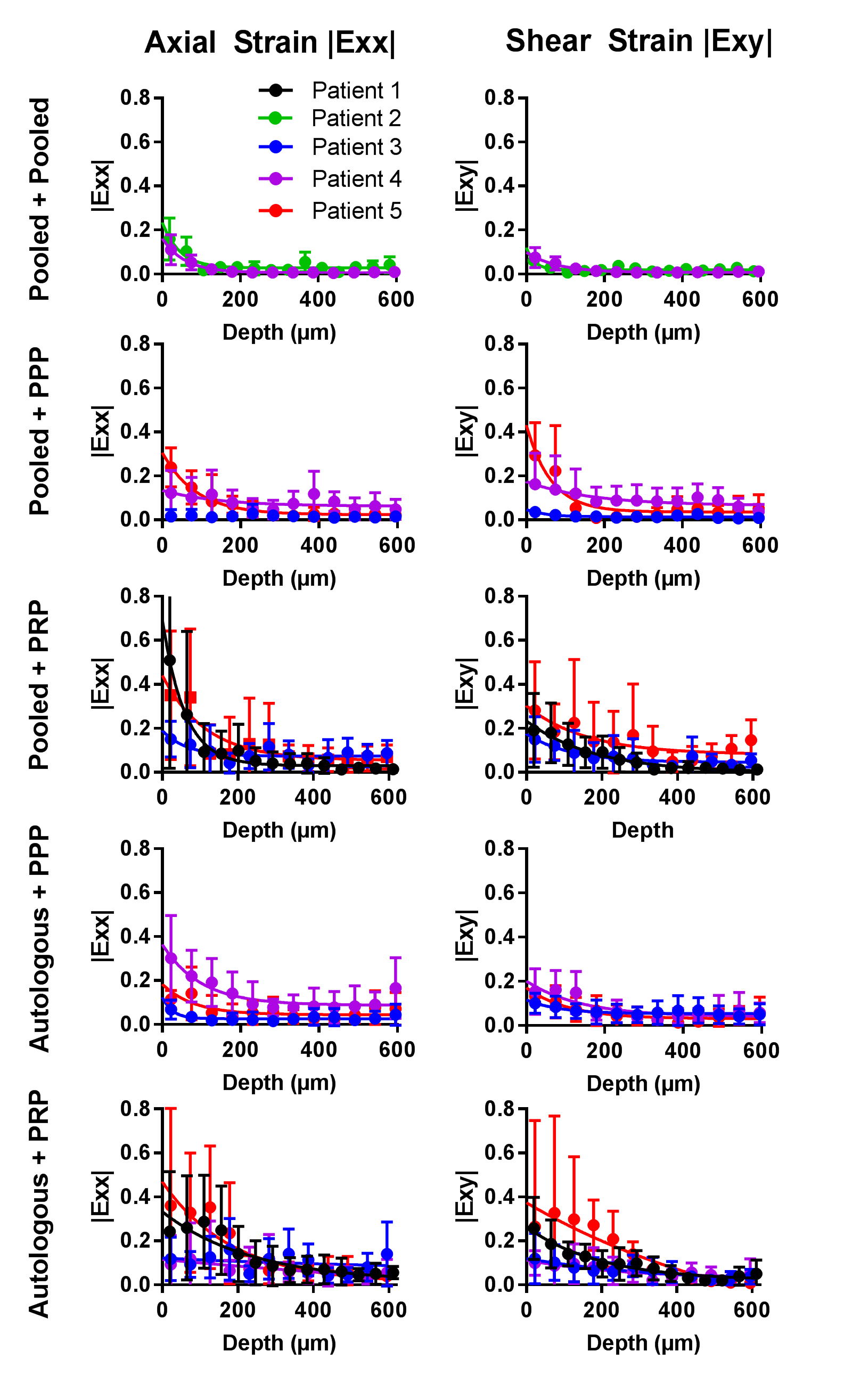

Supplement: S1 Fig — Column header denotes strain and row header denotes sealant group (thrombin + fibrinogen). Exponential decay fits plotted as lines through data points. Color denotes the patient and date tested. Patient 3 in allogeneic thrombin + PPP fibrinogen sealant group was only sample to not converge to an exponential fit as strains were consistent and below 0.03 for all depths. Average strain across 200 μm width region at repair interface ± standard deviations. (TIF) [file pone.0224756.s001.tif]

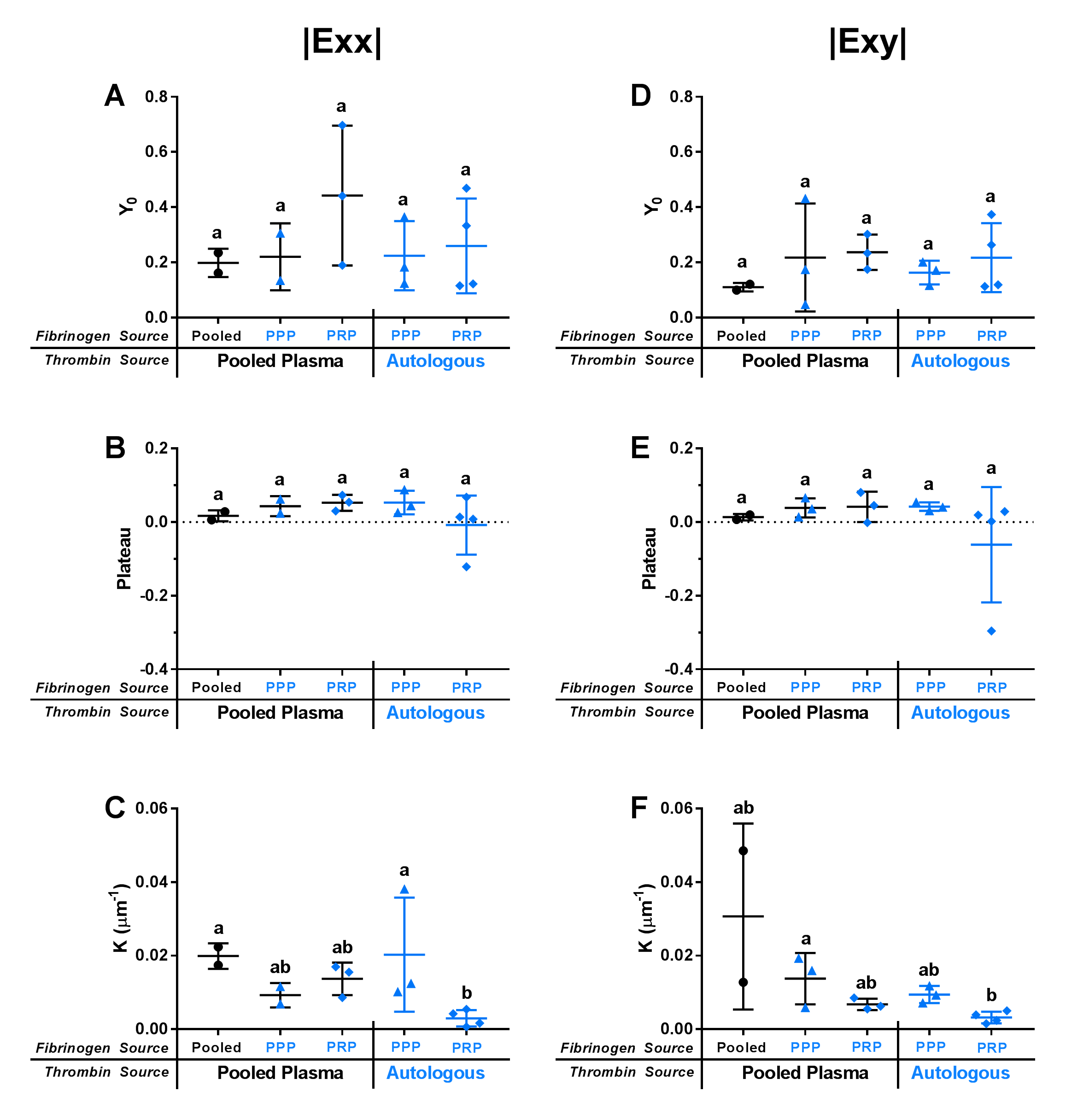

Supplement: S2 Fig — Axial (a-c) and shear strain (d-f) exponential fit parameters from individual samples for all sealants (y0: strain value where depth is 0 μm, plateau: strain value at infinite depths, K: rate constant). The autologous thrombin + PRP fibrinogen sealant had significantly lower K values (rate constants) for both axial and shear strains. Letters denote statistical significance where sealants with same letters were not statistically different and sealants with different letters were statistically significant. (TIF) [file pone.0224756.s002.tif]
