# Supplementary material for: The clot thickens: Autologous and allogeneic fibrin sealants are mechanically equivalent in an ex vivo model of cartilage repair
Source: PLoS One. 2019 Nov 8;14(11):e0224756. doi: 10.1371/journal.pone.0224756 (PMC6839864; doi:10.1371/journal.pone.0224756)
Supplement: S1 Supporting Information — Detailed methods for thromboelastography and thrombin clotting time assay measurements conducted in this study. Additional information on how box cox transformations were performed on data for statistical analyses. (DOCX) [file pone.0224756.s004.docx]

**Supporting information - Thromboelastography and Thrombin Clotting Time Assays Methods**

*Thromboelastography (TEG)*

A thromboelastograph (TEG®, Haemonetic Corporation, Braintree, MA) was used to monitor the viscoelastic properties of fibrin clots formed from recalcified citrate anticoagulated PPP and PRP samples (n=3) activated with a human recombinant tissue factor reagent (Innovin, Siemens Inc., Marburg, Germany); allogeneic fibrinogen was too viscous for this assays. The assays were performed based on previous studies developing TEG techniques to evaluate hemostasis[1,2]. The TEG parameters generated by the instrument software include initiation (R) and rate of fibrin formation (K, angle), maximal clot strength (MA), tensile properties over time (G), extent of lysis at 30 minutes and 60 minutes (LY30, LY60) and time to maximal lysis (CLT).

PPP had a longer R (time to initial fibrin formation, p<0.05) and K (time to achieve clot strength at an amplitude of 20 mm, p<0.05), but lower angle (rate of clot formation, p<0.05), MA (p<0.05), and G (p<0.05) compared to PRP (Table S-1, paired t-tests). There were no differences in time measures of fibrinolysis between autologous fibrinogen sources (p=0.40 for LY30, p=0.54 for LY60, and p=0.24 for CLT).

*Thrombin Clotting Time Analysis*

Modified thrombin clotting time tests (TCT) were used to compare the rate of fibrin formation in a human reference plasma reacted with a bovine thrombin reagent containing a defined thrombin potency versus allogeneic or autologous thrombin sources (n=5-6). Briefly, the TCT endpoint was detected using a manual tilt-tube method[3] in an assay configured with 100 µL of a human standard plasma (FACT, George King Biomedical, Overland Park, KS) warmed to 37˚C. The time to clot formation was then recorded after the addition of 100 µL of a bovine thrombin reagent (12.5 IU/mL thrombin, Triniclot fibrinogen, Triniclot, Wicklow, Ireland).

Allogeneic thrombin induced clotting faster than autologous source thrombin (p<0.001, t-test) with an estimated allogeneic thrombin activity of 770±84 IU/mL (mean ± SD). This value is similar to the manufacturer’s reported value of 400-625 IU/mL. In contrast, the thrombin activity of autologous thrombin was two orders of magnitude lower at 3.8±2.7 IU/mL.

**Supporting information - Box Cox Transformations**

For pull apart results and strain exponential decay fit parameters, Box-Cox power transformations were used to appropriately transform data if residuals were not normally distributed. For pull apart results, data transformations based on Box-Cox transformations using the R software package were as follows: Young’s Modulus: lambda=0.5, bcPower function; ultimate tensile stress: no transformation; ultimate tensile strain: lambda=0 (log transformation), bcPower function; toughness: lambda=0.33, bcPower function. For strain exponential decay fit parameters, data transformations based on Box-Cox transformations were as follows. For axial strain (E_xx_): Y_0_: lambda=0 (log transformation), bcPower function; plateau: lambda = 1, gamma = 0.1, bcnPower function; K: lambda=0 (log transformation), bcPower function. For shear strain (E_xy_): Y_0_ no transformation; plateau: lambda = 1, gamma = 0.1, bcnPower function; K: lambda = 1, bcPower function. The bcnPower function was used when negative values were present in data set and first transformed the data according to the equation: $x=0.5(U+\sqrt{U^{2}+{gamma}^{2})}$. If data set contained no negative values, no gamma transformation was performed. When lambda was equal to zero the data was log transformed, otherwise the data was transformed according to the equation: $\frac{x^{lambda}-1}{lambda}$.

1. Epstein KL, Brainard BM, Gomez-Ibanez SE, Lopes MAF, Barton MH, Moore JN. Thrombelastography in Horses with Acute Gastrointestinal Disease. J Vet Intern Med. John Wiley & Sons, Ltd (10.1111); 2011;25: 307–314. doi:10.1111/j.1939-1676.2010.0673.x

2. Epstein KL, Brainard BM, Lopes MAF, Barton MH, Moore JN. Thrombelastography in 26 healthy horses with and without activation by recombinant human tissue factor. J Vet Emerg Crit Care. John Wiley & Sons, Ltd (10.1111); 2009;19: 96–101. doi:10.1111/j.1476-4431.2008.00381.x

3. Triplett DA, Harms CS. Thrombin clotting time. In: DA T, CS H, editors. Procedures for the coagulation laboratory. Chicago: American Society of Clinical Pathologists Press; 1981. pp. 38–41.
