# Supplementary material for: The clot thickens: Autologous and allogeneic fibrin sealants are mechanically equivalent in an ex vivo model of cartilage repair
Source: PLoS One. 2019 Nov 8;14(11):e0224756. doi: 10.1371/journal.pone.0224756 (PMC6839864; doi:10.1371/journal.pone.0224756)
Supplement: S1 Table — The TEG parameters generated by the instrument software include initiation (R) and rate of fibrin formation (K, angle), maximal clot strength (MA), tensile properties over time (G), extent of lysis at 30 minutes and 60 minutes (LY30, LY60) and time to maximal lysis (CLT). Bolded p-values are statistically significant. Means ± standard deviations (n = 3). (DOCX) [file pone.0224756.s005.docx]

**S1 Table. Thromboelastography results for autologous fibrinogen sources.**

| Fibrinogen Source | R (min) | K (min) | Angle (°) | Maximum Amplitude (mm) | G (d/sc) | LY30 (%) | LY60 (%) | CLT (min) |
| --- | --- | --- | --- | --- | --- | --- | --- | --- |
| PPP | 2.6±0.6 | 1.7±0.3 | 66.6±3.4 | 55.3±2.3 | 6200±600 | 0.3±0.6 | 2.9±2.8 | 60.4±0.4 |
| PRP | 1.4±0.4 | 0.8±0.0 | 82.8±1.7 | 76.5±4.2 | 16733.3±3927.3 | 5.8±8.5 | 9.7±13.8 | 61.7±0.9 |
| p-value | **0.046** | **0.037** | **0.018** | **0.023** | **0.049** | 0.398 | 0.535 | 0.236 |
